# Computational Design of Auxotrophy-Dependent Microbial Biosensors for Combinatorial Metabolic Engineering Experiments

**DOI:** 10.1371/journal.pone.0016274

**Published:** 2011-01-21

**Authors:** Naama Tepper, Tomer Shlomi

**Affiliations:** Department of Computer Science, Technion-IIT, Haifa, Israel; Virginia Tech, United States

## Abstract

Combinatorial approaches in metabolic engineering work by generating genetic diversity in a microbial population followed by screening for strains with improved phenotypes. One of the most common goals in this field is the generation of a high rate chemical producing strain. A major hurdle with this approach is that many chemicals do not have easy to recognize attributes, making their screening expensive and time consuming. To address this problem, it was previously suggested to use microbial biosensors to facilitate the detection and quantification of chemicals of interest. Here, we present novel computational methods to: (i) rationally design microbial biosensors for chemicals of interest based on substrate auxotrophy that would enable their high-throughput screening; (ii) predict engineering strategies for coupling the synthesis of a chemical of interest with the production of a proxy metabolite for which high-throughput screening is possible via a designed bio-sensor. The biosensor design method is validated based on known genetic modifications in an array of E. coli strains auxotrophic to various amino-acids. Predicted chemical production rates achievable via the biosensor-based approach are shown to potentially improve upon those predicted by current rational strain design approaches. (A Matlab implementation of the biosensor design method is available via http://www.cs.technion.ac.il/~tomersh/tools).

## Introduction

In recent years, metabolic engineering has emerged as a discipline that utilizes modern genetic tools for the construction of organisms capable of fuel and chemical production. Metabolically engineered microbial strains are now being used in the industry for the production of various chemicals [1], [2], while significant ongoing efforts are made to engineer microbes to synthesize additional chemicals of interest [3], [4], [5], [6]. The engineering of microbial metabolism follows two paradigms: (i) *A rational design approach* - focused on the engineering of cellular phenotypes using rational modifications (typically gene additions, deletions, up and down regulation, etc) based on existing stoichiometric, kinetic, and regulatory knowledge of a system [7], [8]. (ii) *A combinatorial approach* - generating genetic diversity in a population followed by screening and selection for improved phenotypes [9]. This approach is sometimes followed by *inverse metabolic engineering* (IME), which aims to discover the genetic factors that confer the phenotype and transfer them to another strain by directly applying these genetic modifications [10].

Computational modelling in metabolic engineering has traditionally been used to rationally design the effect of genetic modifications on metabolism. However, such modeling approaches commonly involve either kinetic analysis [11] which requires detailed enzyme kinetic information that is still mostly unknown, or Metabolic Control Analysis [12] that requires experiment-based measurements of flux control coefficients that are also mostly unavailable. An alternative modeling approach, called constraint-based modeling (CBM), analyzing the function of genome-scale metabolic networks through relying solely on simple physical-chemical constraints[13], [14]. Such genome-scale network models are currently available for a variety of microorganisms [15], [16], [17], [18], [19]. Various CBM methods focus on different types of genetic manipulations that can be performed by engineering microbial strains, including gene knockouts (OptKnock and RobustKnock), gene additions (OptStrain), and up- and down-regulation of metabolic enzymes (OptReg and OptForce) [20], [21], [22], [23], [24]. However, although rational design of genetic strategies for chemical production has been successful in some applications (see [25], [26] for reviews), in many cases, the sheer complexity of biological networks and simplifying assumptions that underlie current methods lead to inexact predictions.

The combinatorial approach for metabolic engineering via random mutagenesis followed by screening for specific phenotypes of interest, has long been the gold standards for strain improvement in industry. A variety of recombinant DNA techniques are available for generating random genetic changes, thus introducing the possibility of uncovering regulatory, kinetic, or unknown/poorly understood targets not encompassed in current models (see [9] for a review). This approach is commonly used for functional genomics and phenotypic engineering [27], [28], and has been shown to be remarkably successful for the case of lycopene production in *E. coli* [29]. However, while much progress has been made in the development of experimental techniques for generating combinatorial strain diversity, efficient methods to perform high-throughput screening for chemical producing strains are still lacking. Indeed, a major hurdle with this approach is that many chemicals do not have easy to recognize attributes, making the process of identifying their secretion (using common screening methods such as GC-MS) expensive and time consuming. Pfleger et al. related to this issue, claiming that “Combinatorial strategies are only as good as the screens used to distinguish individual library members” [30]. This problem has led previous research to focus mainly on the production of easily recognizable chemicals, such as pigments that can be detected based on color discrimination [25].

To address the challenge of performing high-throughput screening for chemical production in combinatorial engineering experiments, the usage of microbial bio-sensors for small molecules was suggested [30], [31]. Auxotrophy-dependent microbial biosensors are engineered strains that are auxotrophic to a chemical of interest and hence can be used to detect and quantify the concentration of a chemical in the environment. *E. coli* based biosensors have been previously constructed for vitamins [32] and various amino-acids [30], [33], [34], [35], [36]. Pfleger et al. describe a manual design of an *E. coli* strain, auxotrophic to mevalonate that expresses a green fluorescent protein and reports on the mevalonate concentration in the growth medium through a change in growth rate. In brief, the method involves the generation of a random library of potential producer strains of mevalonate grown in separate cultures, then they are removed via centrifuge and their spent media is moved to new cultures inoculated with the biosensor for mevalonate. The biosensor's growth under each spent media culture reflects the concentration of mevalonate, secreted earlier by a producer strain.

**Figure 1:**
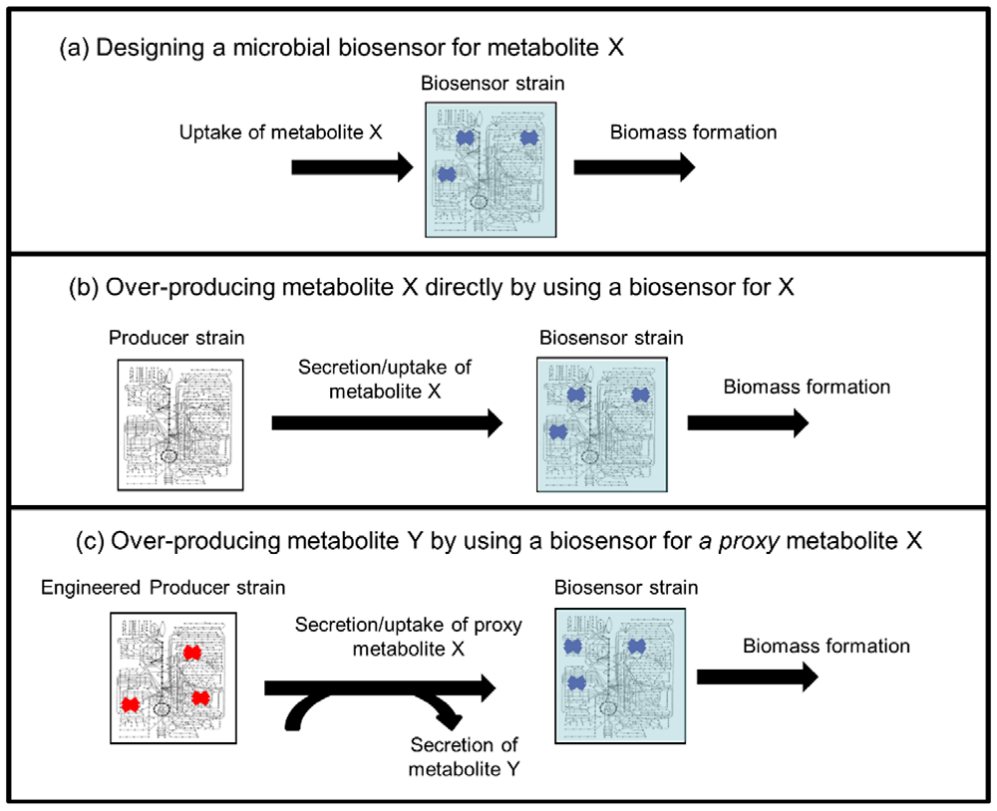
A schematic illustration of metabolite over-production strategies based on microbial biosensors. (a) The first step involves the design of a microbial biosensor whose growth depends on the presence of some metabolite, denoted by X, in the growth medium. (b) The designed biosensor can be directly used within combinatorial metabolic engineering experiments to perform high-throughput screening for producer strains that secrete chemical X. (c) Here, we suggest that a biosensor for chemical X can be used within combinatorial metabolic engineering experiments to over-produce a different chemical of interest, denoted by Y, whose production is coupled to the secretion of X (based on rationally designed genetic manipulations in the producer strain).

This paper presents a novel approach for rationally designing microbial biosensors that can be used within combinatorial experiments towards the production of chemical of interest. Specifically, we present computational methods to (i) design microbial biosensors for chemicals of interest based on substrate auxotrophy (Figure 1a) that can be used to perform high-throughput screening for their production (Figure 1b). (ii) Predict engineering strategies for coupling the synthesis of a chemical of interest with the production of a proxy metabolite for which high-throughput screening is possible via a designed bio-sensor (Figure 1c). We show that predicted chemical production rates achievable via the biosensor-based approach may potentially improve upon those achievable via current rational design methods.

## Results

### Computational design of microbial biosensors based on substrate-auxotrophy

Given a metabolic network model for some microbial species, a chemical to be sensed denoted by *C*, and a definition of a growth medium denoted by *M*, the biosensor design method aims to search for genetic modifications such that: (i) The bio-sensor strain would grow in a medium that consists of at least nutrients that are in *M* and *C*, and (ii) would not grow in a medium where *C* is absent, even if it consists of all metabolites (other than *C*) that can be transported into the biosensor strain. Notably, the common definition of substrate-auxotrophy is less restrictive than the above, and in many cases, strains considered auxotrophic for a chemical *C*, may actually also grow in the absence of *C*, utilizing a different substrate as input to synthesize *C*. We hence refer to a microbial strain that realizes the above growth constraints as being *ultra-auxotrophic* for chemical *C*. A microbial strain that is *ultra-auxotrophic* for *C* (considering some medium *M*), can be used as a biosensor within combinatorial metabolic engineering experiment, to detect and quantify the concentration of *C* (similarly to the experiment performed by [30]). Specifically, in these experiments, after the producer strains are grown, their spent media is moved to new cultures, where medium *M* is added, and the biosensor strains are grown. The ability of the biosensor to grow indicates whether chemical *C* is present in the spent medium (i.e. secreted from the producer strain), with the total biomass of the grown biosensor strain reflecting the concentration of *C* (as further explained below). The requirement for ultra-auxotrophy guarantees that the biosensor strain would not grow in case *C* is absent from the spent media, regardless of which additional chemicals were potentially secreted by the producer strains.

**Figure 2:**
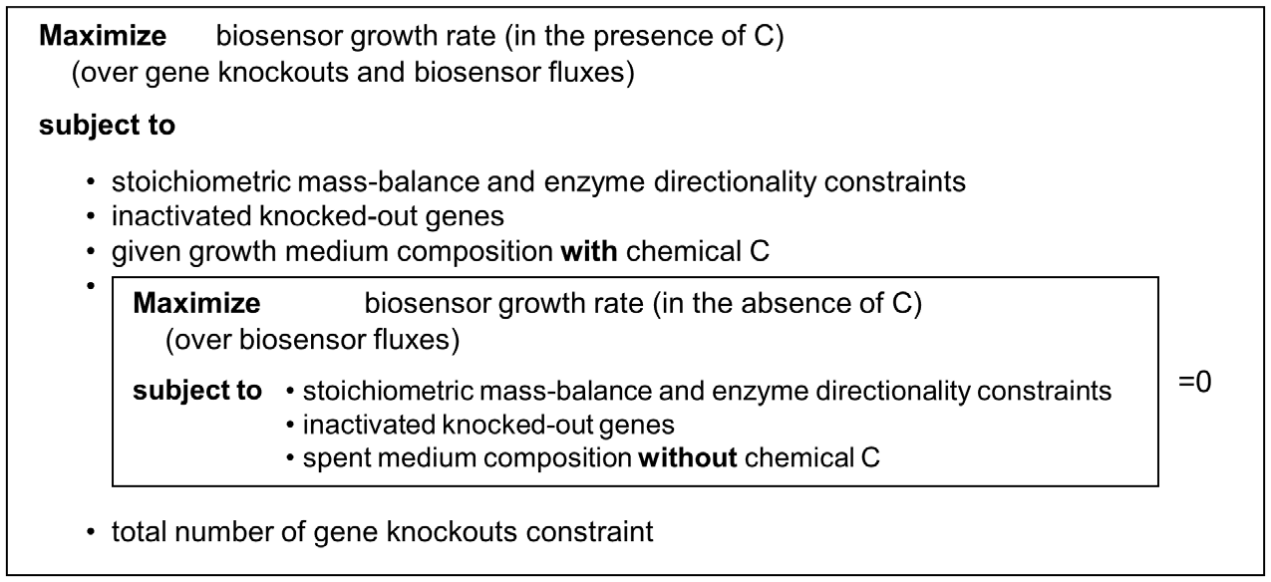
A schematic representation of the bi-level optimization problem that underlies the biosensor design method. The outer optimization problem searches for a set of gene knockouts and a feasible flux distribution with maximal growth rate when chemical *C* is present in the growth medium. The inner optimization problem is used to enforce a maximal growth rate of zero (reflecting no growth) when *C* is absent from a rich growth medium.

The search for a set of gene knockouts that would give rise to ultra-auxotrophy is performed via a bi-level optimization problem, considering stoichiometric mass-balance, reaction directionality, and knockout constraints (i.e. zero flux through knocked-out reactions; Methods) (Figure 2). This optimization is formulated as a mixed-integer linear programming (MILP) problem [20], [24], which is optimally solved in a manner of seconds to minutes on a standard PC. Notably, while previous studies have already employed bi-level and MILP optimizations for various purposes in the context of metabolic network analysis [20], [21], [37], [38], [39], this is the first application of this approach for the design of auxotrophic biosensors – and specifically for the search of gene knockout combinations that would give rise to different growth phenotypes under two different growth media.

**Figure 3:**
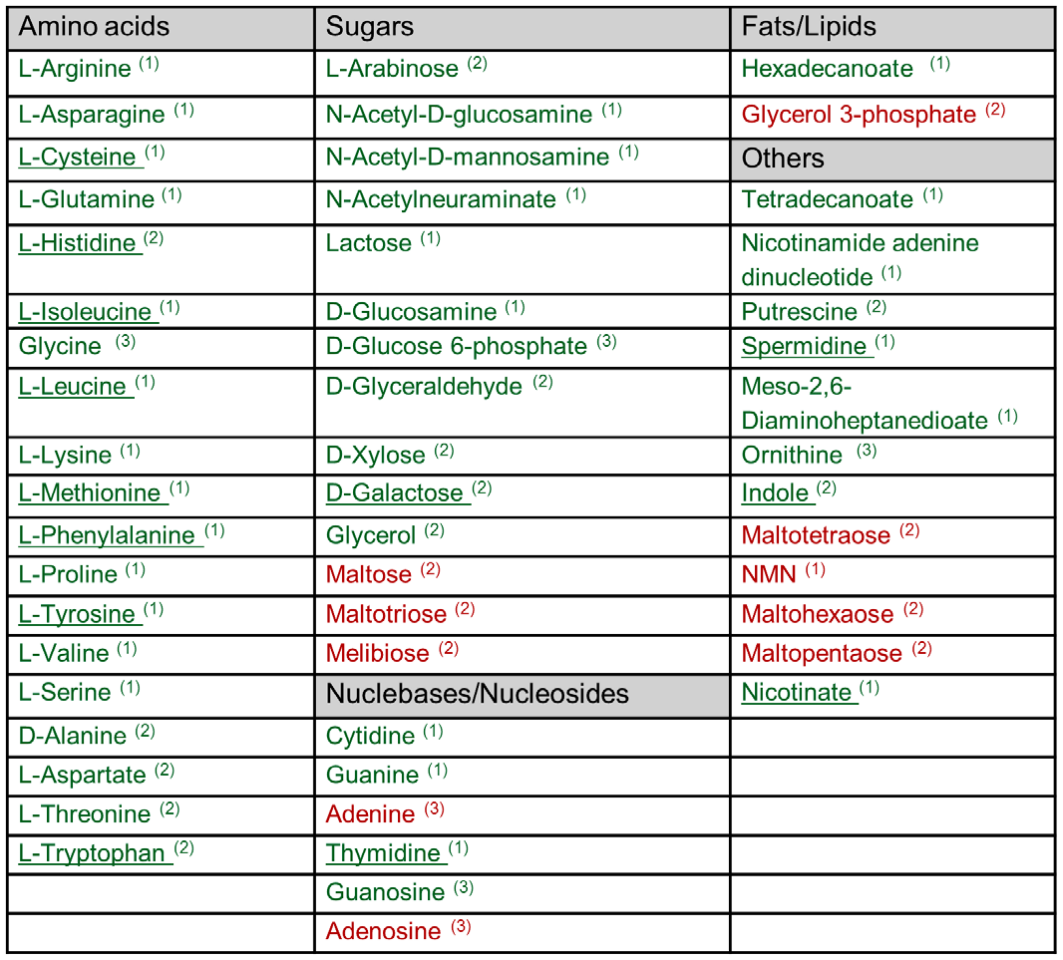
A list of metabolites with predicted biosensor designs. The 43 biosensors predicted under glucose minimal medium are marked in green, while the additional 10 biosensors predicted under a rich medium are colored red. Biosensors whose biomass production yields are predicted to be insensitive to the exact metabolite-composition of the spent medium are underlined. The number of gene knockouts predicted for the various biosensors is shown in superscript.

We applied the above method on a genome-scale metabolic network model of *E. coli* metabolism iJR904 [18], consisting of 143 different chemicals that have a transport reaction in the model, allowing their potential uptake from the growth medium. The network model includes 904 metabolic enzyme-coding genes, accounting for 1075 reactions and 761 metabolites. We considered two possible growth media for the biosensor strains: a glucose minimal medium and a rich medium (consisting of all metabolites that may potentially be taken up by *E. coli*, other than the sensed chemical). The latter medium, in which a large number of nutrients are expected to be manually added to the spent media is more complex from an experimental perspective, though as shown below, it increases the number of designed biosensors and enables to better quantify the concentration of sensed chemicals. We restricted the analysis to allowing up to a total of three knockouts. When considering a minimal medium, we predict biosensor designs for 43 chemicals, and when considering a rich medium, we predict additional biosensor designs for 10 chemicals (Figure 3). The predictions include biosensors for 19 amino acids and 14 sugars among others. Each predicted biosensor strain is expected to grow in a spent medium (supplemented with either glucose minimal or rich medium) in the presence of its sensed chemical, and not grow in its absence, regardless of which additional chemicals were potentially secreted by the producer strain. The specific details of the predicted knockout combinations for each bio-sensor are shown in Table S1.

**Figure 4:**
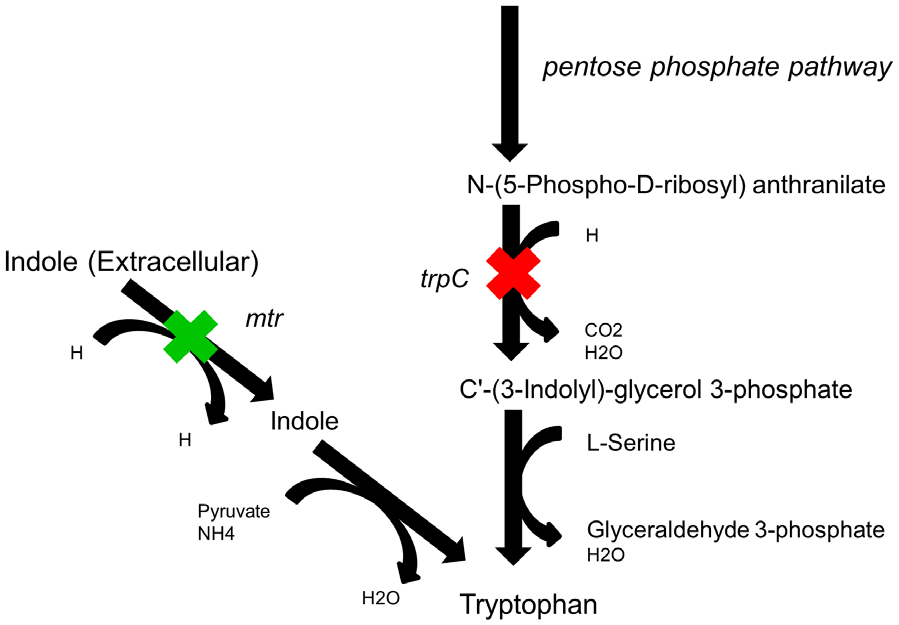
Gene knockouts that are predicted to give rise to a *tryptophan* biosensor. The known *E. coli* auxotrophic to tryptophan has trpC gene knocked-out (marked with a red X), blocking the synthesis of tryptophan from an intermediate metabolite in the pentose phosphate pathway. Our predictions show that if only this gene is knocked-out then *E. coli* may be able to utilize indole as a substrate to produce tryptophan, suggesting that a biosensor strain whose growth should depend specifically on the presence of tryptophan should hence also have its indole-to-tryptophan pathway knocked-out (marked with a green X).

Comparing the predicted amino-acids biosensors with a list of known amino-acid auxotrophic strains (extracted from the Coli Genetic Stock Center (CGSC); http://cgsc.biology.yale.edu/) shows a good match between the two (Table 1). Specifically, for 10 amino-acids, the predicted knockouts match those of the known auxotrophic strain. For 5 amino-acids the predicted knockouts differ from the ones described in the CGSC database, though further literature search revealed experimental evidence showing that all predicted knockouts also lead to the desired auxotrophy [40], [41], [42]. Simulating the knockouts that give rise to these 5 auxotrophic strains in CGSC showed that they indeed lead to substrate auxotrophy, though not to the desired ultra-auxotrophy – i.e. these strains should also be able to grow under rich media, even in the absence of the corresponding metabolites in the medium. For example, for the tryptophan biosensor, we predicted the double knockout of indole-3-glycerol-phosphate and indole transporter, while the known CGSC auxotroph consists only of the former knockout (Table 1). Our analysis shows that if only 3-glycerol-phosphate synthase enzyme is knocked-out then *E. coli* should be able to utilize indole as a substrate to produce tryptophan (Figure 4). Notably, the double knockout predicted by our method was previously implemented by [43] and was shown to be able to grow on rich media, though, its ability of grow on a media containing only glucose and indole was not tested. For 3 additional amino-acids, the knockouts of the CGSC auxotrophic strains are falsely predicted not to lead to substrate auxotrophy, though in 2 of the cases, the knockout of an additional reaction does lead to auxotrophy predictions. I.e., in the later cases, the model over-predicts the number of modifications required to guarantee the desired ultra-auxotrophy. The latter false predictions of gene knockouts required to achieve auxotrophy may result from genetic down regulation of these genes that is not explicitly accounted for in the metabolic network model.

**Table 1:** A comparison between predicted amino-acid biosensor designs and a list of known amino-acid auxotrophic strains.

	Chemical	Known knockouts leading to auxotrophy	Predicted knockouts leading to ultra-auxotrophy
**MATCH BETWEEN KNOWN AND PREDICTED KNOCKOUTS**	**Cysteine**	serine O-acetyltransferase	serine O-acetyltransferase
	**Lysine**	diaminopimelate decarboxylase	diaminopimelate decarboxylase
	**Methionine**	homoserine O-succinyltransferase	homoserine O-succinyltransferase
	**Tyrosine**	prephenate dehydrogenase	prephenate dehydrogenase
	**Histidine**	histidinol-phosphatase	histidinol-phosphatase
		imidazoleglycerol-phosphate dehydratase	imidazoleglycerol-phosphate dehydratase
	**Glutamine**	glutamine synthetase	glutamine synthetase
	**Leucine**	3-isopropylmalate dehydrogenase	3-isopropylmalate dehydrogenase
		2-Oxo-4-methyl-3-carboxypentanoate decarboxylation	2-Oxo-4-methyl-3-carboxypentanoate decarboxylation
	**Phenylalanine**	prephenate dehydratase	prephenate dehydratase
	**Serine**	phosphoglycerate dehydrogenase	phosphoglycerate dehydrogenase
	**Asparagine**	asparagine synthetase	asparagine synthetase
		asparagine synthase (glutamine-hydrolysing)	asparagine synthase (glutamine-hydrolysing)
**KNOWN KNOCKOUTS PREDICTED TO LEAD TO AUXOTROPHY, BUT NOT TO ULTRA- AUXOTROPHY**	**Arginine**	cetylornithine deacetylase	argininosuccinate lya
		N-acetylornithine deacetylase	
	**Tryptophan**	indole-3-glycerol-phosphate synthase	indole-3-glycerol-phosphate synthase
		phosphoribosylanthranilate isomerase	Indole transport via proton symport
	**Leucine**	dihydroxy-acid dehydratase (2,3-dihydroxy-3-methylbutanoate)	2-isopropylmalate synthase
	**Valine**	dihydroxy-acid dehydratase (2,3-dihydroxy-3-methylbutanoate)	acetohydroxy acid isomeroreductase
	**Isoleucine**	dihydroxy-acid dehydratase (2,3-dihydroxy-3-methylbutanoate)	2-aceto-2-hydroxybutanoate synthase
**MISMATCH BETWEEN KNOWN AND PREDICTED KNOCKOUTS**	**Glycine**	glycine hydroxymethyltransferase	glycine hydroxymethyltransferase
			Threonine Aldolase
			L-threonine dehydrogenase
	**Threonine**	threonine synthase	threonine synthas
		4-Hydroxy-L-threonine synthase	Threonine Aldolas
	**Proline**	glutamate-5-semialdehyde dehydrogenase	pyrroline-5-carboxylate reductase

For the first 10 amino-acids (rows in the table), the predicted knockouts match exactly those of the known auxotrophic strains. For the next 5, the known knockouts are correctly predicted as causing auxotrophy (when simulated via FBA in the model), but not to ultra-auxotrophy, while the biosensor design method yielded different knockouts that are predicted to lead to the desired ultra-auxotrophy. For the last 3 amino-acids in the table, the predicted and known knockouts differ.

The predicted biosensors can be used not only to detect the presence of chemicals of interest in a medium, but also to assess their actual concentration based on the total biomass of the grown biosensor strains (which can be easily detected experimentally, e.g., by a utilizing green fluorescent protein, as done in [30]). When rich medium is assumed to be added to the spent medium, the biosensor's biomass yield should not be affected by the potential secretion of additional metabolites by the producer strains (which are ‘masked’ by the manual addition of all metabolites that may be taken up by *E. coli*). Hence, in this case, the concentration of the sensed chemical can be accurately determined based on the total biomass of the grown biosensor strain. When only glucose minimal medium is assumed to be added to the spent media, the biosensor's biomass yield may change depending on the secretion of additional chemicals by the producer strain. For example, the xylose biosensor achieves a significantly higher biomass yield when additional metabolites (other than xylose) not included in glucose minimal media are present in the media (Figure S1a). On the other hand, the galactose biosensor's biomass yield does not change when additional metabolites (not present in glucose minimal medium) are added to the spent media (Figure S1b). Overall, out of the 43 predicted biosensors for glucose minimal media, the biomass yield of 13 was found to be insensitive to the specific metabolite composition of the spent media (marked with an underline in Figure 3). The remaining biosensors may not provide an accurate estimation of chemical concentrations under glucose minimal media, though the latter can still be achieved by growing the biosensors on a rich media. Notably though, the higher biomass yields achieved in rich media may limit chemical detection ranges due to practical consideration involving excess biomass production of the biosensor strain (while the later may be tuned experimentally by lowering the amount of secreted chemical by the producer strain, by lowering the amount of nutrients given in its growth medium).

### Over-producing chemicals of interest using the designed biosensors

To demonstrate the applicability of the predicted biosensors in over-producing chemicals of interest via combinatorial metabolic engineering experiments (such as that performed by [30]), we compare the optimal possible outcome of such experiments with that achievable by existing rational design approaches. Since combinatorial experiments utilize biosensors to iteratively select producer strains with high chemical production yields, the maximal possible production rate achievable by this approach is computed via FBA (Methods). Similarly, previous studies have shown that FBA is able to correctly predict the maximal possible biomass production rate following adaptive evolution of *E. coli* [44], [45], [46]. Notably, in practice, purely combinatorial experiments may not necessarily reach the maximal production rate predicted by FBA, the latter requiring further metabolic engineering interventions.

**Figure 5:**
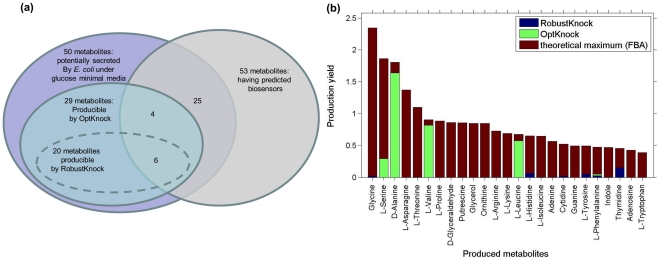
Chemical production via rational design versus the biosensor-based combinatorial approach. (a) A Venn diagram showing the overlap between the set of metabolites which have predicted biosensors, the set of metabolites that may potentially be produced and secreted by *E. coli* under glucose minimal media, and the sets of metabolites whose over-production can be rationally designed via OptKnock and RobustKnock. Out of a set of 25 metabolites that may potentially be produced by *E. coli* and have a predicted biosensor (which can be used in combinatorial engineering experiment to over-produce them), only 10 metabolites can also be over-produced by OptKnock or RobustKnock. (b) The achievable over-production yields of the 25 metabolites predicted by OptKnock and by RobustKnock versus the maximal theoretical yield potentially achievable via the biosensor-based approach (as predicted via FBA).

Here, we considered combinatorial experiments that utilize *E. coli* also as the producer organism, aiming to over-produce various chemicals that have predicted biosensors (from the above section). FBA is applied to predict the maximal theoretical production yield of a chemical of interest, while enforcing a minimal growth rate of 0.1 mmol/gr(DW)h to maintain viability (the same threshold used in the implementation of OptKnock and RobustKnock; other choices of this threshold gave quantitatively similar results). The results show that combinatorial metabolic engineering experiments aiming to over-produce chemicals using the predicted biosensors would enable to produce 25 metabolites with a rate greater than zero (Figure 5a). These include important metabolites for industrial purposes, such as indole (used in the perfume industry) and glycerol (widely used in pharmaceutical industry).

We compared these predicted combinatorial-biosensor results to those that may be achieved using two leading rational design methods, OptKnock [20] and RobustKnock [24], for predicting gene knockout strategies for over-producing the same chemicals using *E. coli*. OptKnock works by searching for a gene knockouts combination that couples the production and secretion of a chemical of interest with the production of biomass (and hence with the bacterial growth rate). RobustKnock is a robust variant of OptKnock that searches for knockout combinations that maximizes the minimal (guaranteed, based on the model constraints) secretion rate of the chemical, considering the presence of the alternative pathways in a metabolic network that may prevent the organism from reaching the maximal theoretical chemical production rate predicted by OptKnock (Methods). For both OptKnock and RobustKnock were allowed up to three concurrent gene knockouts, in accordance with the number of knockouts considered in the biosensor designs.

The application of OptKnock predicted gene deletions strategies that enable the production of only 10 chemicals out of the 25 metabolites whose production is achievable via biosensors. The application of RobustKnock shows that a non-zero secretion of only 6 of the 25 metabolites can be guaranteed (considering the model constraints) by coupling their production with biomass formation. Furthermore, the maximal possible production yields predicted by OptKnock and RobustKnock for these chemicals are significantly lower than the optimal yields that may theoretically be achieved by the biosensor-based approach (Figure 5b), testifying for the potential added value of the latter approach over current rational design methods. Notably, while our application of OptKnock and RobustKnock was limited to no more than three concurrent knockouts, previous studies have shown that in many cases, higher order knockouts still do not lead to optimal production yields [47], [48]. For example, in the case of glycerol, allowing up to 10 concurrent gene knockouts has led to a production yield of only 66% of the maximal theoretical yield, compared to 89% achieved by directly using a biosensor for glycerol (see Table S2 and S3 for details). For L-Serine allowing 10 concurrent knockouts has still led to zero guaranteed production yield using rational design.

### Over-producing chemical of interest using biosensors to screen for proxy metabolites

As described above, the predicted biosensors can be used in combinatorial metabolic engineering experiments to directly screen for strains that over-produce 25 out of a total of 50 metabolites producible by *E. coli* under glucose minimal medium. To over-produce the remaining 25 metabolites, we suggest a novel approach, this time focused on engineering the producer strain (rather than the detecting, biosensor strain) via gene knockouts to couple the production of a metabolite of interest with the production of a proxy metabolite for which a biosensor design has already been found (and hence can be used to maximize the production rate of this proxy metabolite; Figure 1c). The prediction of an engineering strategy for the producer strain is facilitated via variants of OptKnock and RobustKnock (referred to as OptKnock-proxy and RobustKnock-proxy), aiming to couple the production rate of a chemical of interest with the production of a given proxy metabolite (instead of coupling it with biomass formation; Methods). OptKnock-proxy's predictions reflect the maximal theoretical production yield of a chemical of interest, assuming a maximal secretion rate of the proxy metabolite. RobustKnock-proxy's predictions reflect the maximal guaranteed secretion rate of a chemical of interest (considering alternative pathways in the metabolic network), assuming a maximal production rate of the proxy metabolite. Both OptKnock-proxy and RobustKnock-proxy additionally consider a lower bound on biomass production rate, to maintain viability, similarly to OptKnock and RobustKnock.

**Figure 6:**
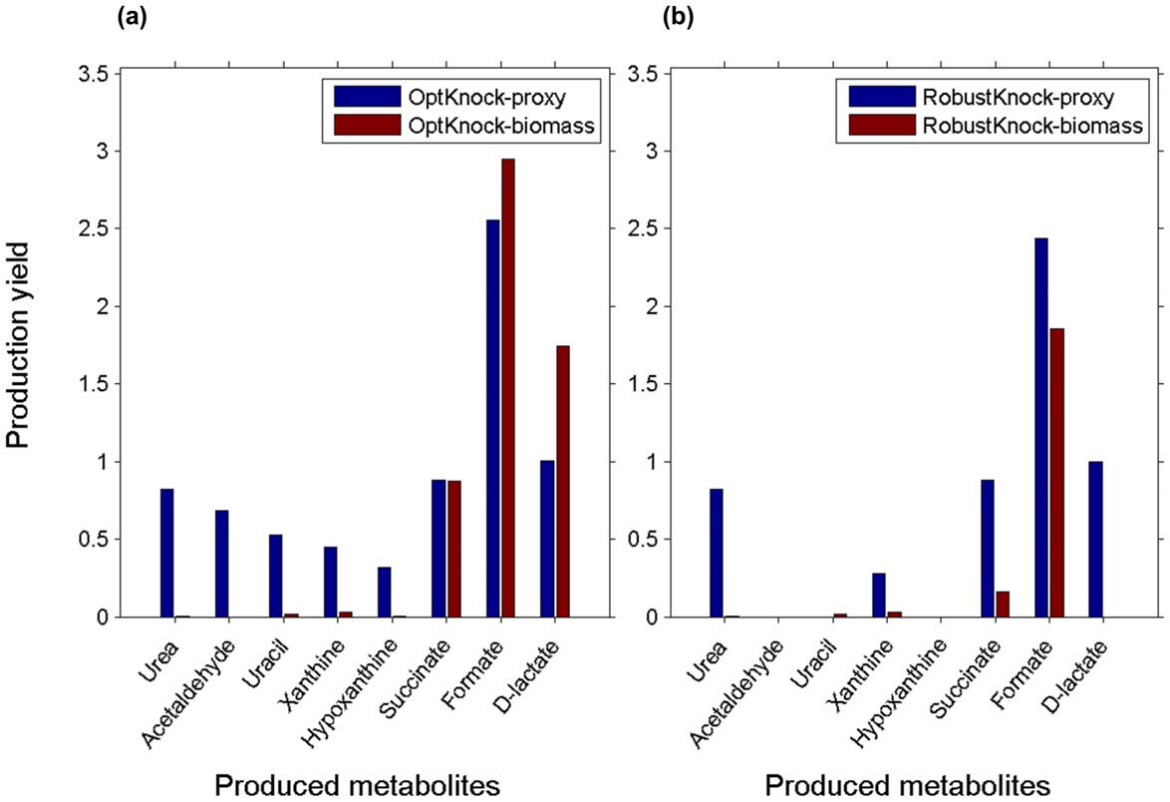
Chemical production via rational design versus the biosensor-proxy approach. Maximal chemical production yields predicted by the biosensor-based methods OptKnock-proxy and RobustKnock-proxy (achieved with one of the 25 designed biosensors), compared with those predicted by the rational design methods, OptKnock and RobustKnock, which couple chemical production rate with biomass.

**Figure 7:**
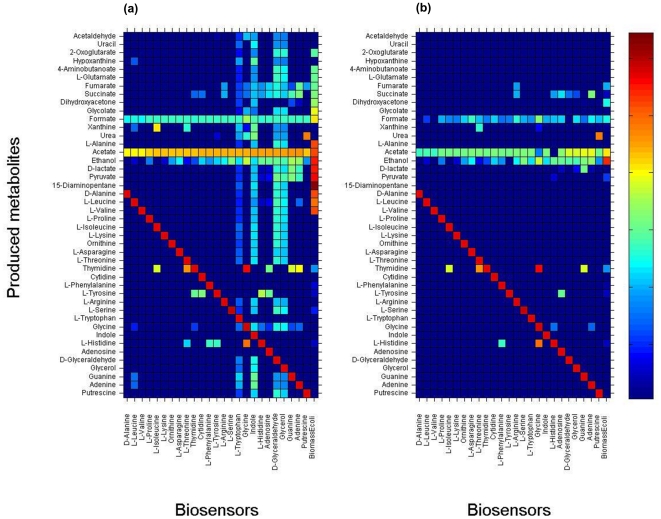
Chemical production yield via the biosensor-proxy approaches utilizing each of the 25 designed biosensors. (a) Maximal chemical production yields predicted by OptKnock-proxy using each of the 25 designed biosensors. (b) Minimal, guaranteed chemical production yield predicted by RobustKnock-proxy using each of the designed biosensors. Blue table entries denote zero metabolite production yields while red entries denote maximal theoretical production yields (as predicted via FBA). The rightmost columns represent the predicted over-production rates computed by OptKnock (a) and RobustKnock (b), by coupling chemical productions to biomass production.

We applied OptKnock-proxy and RobustKnock-proxy to predict gene knockouts that would enable the coupling the production of each of the 25 metabolites for which no biosensor design is available with each of the 25 metabolites that do have a predicted biosensor design as a potential proxy (Table S3). We find that OptKnock-proxy predicts knockout strategies that enable the production of 20 out of the 25 metabolites (which have no predicted biosensor of their own), utilizing at least one out of the 25 predicted biosensors. Comparing the predicted production yields with those achievable by rational design, via OptKnock (which aims to couple the chemical production with biomass formation), shows higher potential production yields for OptKnock-proxy for 5 out the 20 metabolites (Figure 6a; Table S3). Applying RobustKnock-proxy predicts knockout strategies for guaranteed, over-production of 11 metabolites, with markedly higher production yields in 5 cases than those predicted by the standard RobustKnock method (Figure 6b; Table S3). Interestingly, we find that knockout combinations predicted by OptKnock-proxy and RobustKnock-proxy (for the entire set of chemicals and proxy metabolites) spans a high number of 86 different knocked-out reactions. Figure 7 shows which proxy metabolites were predicted to facilitate the over-production of various chemicals by OptKnock-proxy and RobustKnock-proxy. As shown, for each chemical whose production is enabled via OptKnock-proxy and RobustKnock-proxy (without a direct sensore), we find that an average number of 6.95 and 3.31 different biosensors, respectively, could lead to its production in a rate higher than zero. We find that several ‘hub’ biosensors, tryptophan, indole, glycerol and glyceraldehyde, enable OptKnock-proxy to design the over-production of a high number of metabolites (Figure 7a). On the other hand, we find that the usage of these hub biosensors cannot guarantee the over-production of many metabolites based on RobustKnock-proxy predictions, with a variety of different biosensors required for guaranteeing the over-production of various metabolites (Figure 7b).

## Discussion

In this paper, we describe a computational method for designing microbial biosensors that can be used to perform high-throughput measurements of the concentration of various small molecules. Applying our method to design biosensors based on *E. coli*, we predict gene knockout strategies for deriving *E. coli*-based sensors for 53 metabolites. The utilization of these biosensors to screen for *E. coli* strains that over-produce the corresponding chemicals is predicted to potentially improve achievable production rates, compared to those predicted by current rational design methods, OptKnock and RobustKnock. Furthermore, we present a new approach for over-producing metabolites by performing gene knockouts that would couple their production and secretion with the production of a proxy metabolite that does have a biosensor (and hence its production rate can be maximized via standard metabolic engineering techniques). The latter approach is predicted to improve the potential production and secretion rates of several metabolites.

A major limitation of current constraint-based rational design approaches in metabolic engineering is that they all aim to couple the production of a chemical of interest with biomass formation, such that evolutionary pressure towards growth rate maximization would lead to increased chemical production rates [20], [21], [22], [24]. A recent work by Ranganathan et al., addresses this issue by suggesting a novel method, OptForce, that does not rely on a definition of a cellular objective function, though it requires additional experimental flux measurements as input [23]. The presented biosensors-based approach addresses the same problem and similarly, does not rely on a coupling between growth rate and chemical production. Notably, while here, our biosensor design method was applied to predict knockout strategies that are limited to no more than three concurrent knockouts, running this method on powerful computer-clusters would enable the prediction of four and even five concurrent knockouts [49], [50]. An additional scale up in terms of number of concurrent knockouts could be achieved by applying heuristic search methods, such as those employed in OptGene [48]. Whereas various metabolic engineering mechanisms may be used in the design of microbial biosensors, the approach described here focuses strictly on the prediction of knockout strategies. A natural extension of the described method could account for additional genetic manipulations in the form of gene up- and down-regulation [22] and gene addition [21]. The latter manipulations are of a particular interest, and may potentially extend the repertoire of metabolites that can be sensed by a microorganism to metabolites that are not taken up by the wild-type strain.

To demonstrate the applicability of the biosensor design method and OptKnock-proxy and RobustKnock-proxy, we chose to focus on *E. coli* (grown under standard glucose minimal media), which is a common target of metabolic engineering experiments and has a highly accurate metabolic network model. Considering the growing interest computational approaches in metabolic engineering and the current rapid reconstruction of metabolic networks (giving rise to more than 50 highly curated metabolic reconstructions published to date [51]), we expect the presented methods, which are obviously general and species independent, to significantly contribute to rational design of combinatorial metabolic engineering experiments with many other of those species in the future.

## Methods

### Constraint-based modelling (CBM) and Flux Balance Analysis (FBA)

*Constraint-based modeling (CBM)* is a mathematical modeling approach for metabolic networks that utilizes knowledge of the network structure together with constraints on its possible behaviors, to predict possible functional metabolic states. Mass-balance constraints imposed by stoichiometry in a chemical network at steady state enforce the sum of all production and consumption rates of each metabolite to be zero:
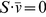
(1)where *S* denotes a stoichiometric matrix in which *S_ij_* corresponds to the stoichiometric coefficient of metabolite *i* in reaction *j*. *S* dimensions are 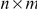, where *n* is the number of metabolites in the network and *m* is the number of reactions. 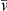 denotes an *m*-dimensional vector of flux rates, where 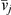 is the flux rate of reaction *j* at steady state. Additional constraints, including those pertaining to the availability of nutrients in the growth media or to the maximal flux that can be supported by specific enzymes can be introduced via the following inequality:
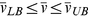
(2)where 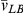 and 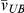 denote lower and upper bounds on metabolic flux rates, respectively. For example, for a substrate uptake flux 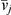, one can set 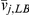 and 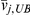 to be equal to the corresponding measured or imposed values. This constraint can also be used to distinguish reversible and irreversible reactions, where 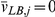 is set for the latter.

Taken together, the constraints limit the allowable functional states of a metabolic network. In mathematical terms, the range of allowable network states is described by a solution space *Φ* that represents the phenotypic potential of an organism. For an underdetermined system, as is typically the case in models of cellular metabolism [26], *Φ* is a convex set in the *m*-dimensional space of fluxes [52]. *Flux Balance Analysis (FBA)* is a particular CBM method that assumes that the network is regulated to maximize a certain cellular function [53], [54]. A natural choice of an objective function in metabolic models of microorganisms is that of biomass maximization [26], [55], as it is reasonable to assume that unicellular organisms have evolved towards maximal growth performance. This process is formalized by introducing a growth reaction (denoted by 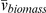) that transforms a linear combination of fundamental metabolic precursors into biomass formation. The presence of alternative pathways in a metabolic network causes FBA to predict a space of feasible flux distributions with a maximal growth rate, rather than a single solution [56].

### A computational method for predicting biosensor designs based on substrate auxotrophy

The biosensor design method is based on a bi-level optimization problem that searches for gene knockouts such that a feasible flux distribution that satisfies stoichiometric mass-balance, reaction directionality, and knockout constraints, will enable high growth rate when chemical *C* is present in the spent media and no growth when it is absent. The problem is formulated as following:
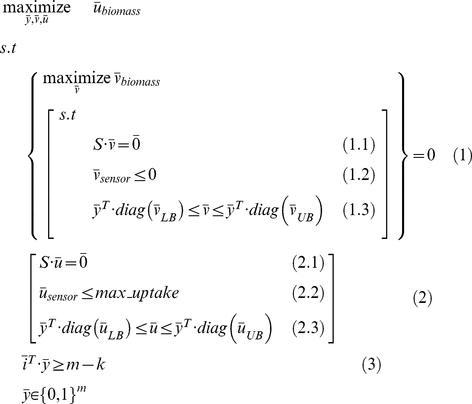
(3)


The outer optimization problem searches for a set of gene knockouts (Boolean variables 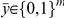) and a feasible flux distribution (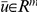) with maximal growth rate when the sensed chemical is present in the growth medium. The inner optimization problem is used to search for a feasible flux distribution 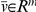 which satisfies the gene knockout constraints when the sensed chemical is absent from the growth medium, enforcing its maximal growth rate to zero. The knockout of the *i*'th reaction is represented by 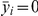 (equations (1.3) and (2.3)), with the total number of concurrent knockouts limited to no more than a pre-defined threshold, *k* (where 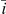 is a vector containing all ones; equation (3)). The symbol 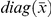 is used to represent a square matrix with 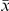 on the diagonal, with all other entries equal to zero.

Stoichiometric mass-balance, enzyme directionality and knockout constraints are enforced for the flux distribution 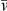 by Equations (1.1) and (1.3) and for 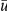 by Equations (2.1) and (2.3), respectively. Lower and upper bounds on flux rates for both 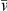 (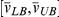) and 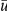
(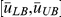) were taken from the metabolic network model of [18]. The upper bounds on exchange reactions for flux distribution *u* enable the uptake of nutrients present in either glucose minimal or rich media. Reaction upper bounds for 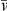 enable the uptake of nutrients present in a rich media (i.e. consisting of all metabolites that may potentially be taken up by *E. coli*). The uptake of the sensed chemical is enabled only for flux distribution 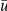 (Equation 2.2), and not for 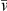 (Equation 1.2). Alternative optimal knockout combinations were obtained using integer-cuts, as described in [21].

## Supporting Information

Figure S1 **The predicted growth rate of the D-xylose (a) and D-galactose (b) biosensors, as a function of sensed chemical update rate, under both poor and rich media.** As shown, the growth yield (represented by the slope of the curve) of the D-xylose biosensor significantly differ between poor and rich spent media. Hence if the D-xylose biosensor is grown after a minimal medium is supplemented to the spent medium, the potential secretion of additional metabolites by the producer strain may affect its biomass yield, leading to inaccurate estimation of the chemical concentration (an accurate estimation of D-xylose concentration can still be achieved by growing the biosensor after adding all nutrients from a rich medium (other than D-xylose, of course) to the spent. On the other hand, the D-galactose biosensor is predicted to have the same biomass yield in both poor and rich medium, facilitating the direct quantification of chemical concentration also in poor medium.
(TIF)

Table S1 **The specific details of the predicted knockout combinations for each bio-sensor.**
(XLS)

Table S2 **Secretion rates of chemical as predicted by OptKnock and RobustKnock.**
(XLS)

Table S3 **Secretion rates of chemical (using different proxies) as predicted by OptKnock-proxy and RobustKnock-proxy.**
(XLS)
