# Supplementary figures and images for: Computational Design of Auxotrophy-Dependent Microbial Biosensors for Combinatorial Metabolic Engineering Experiments
Source: PLoS One. 2011 Jan 21;6(1):e16274. doi: 10.1371/journal.pone.0016274 (PMC3025009; doi:10.1371/journal.pone.0016274)

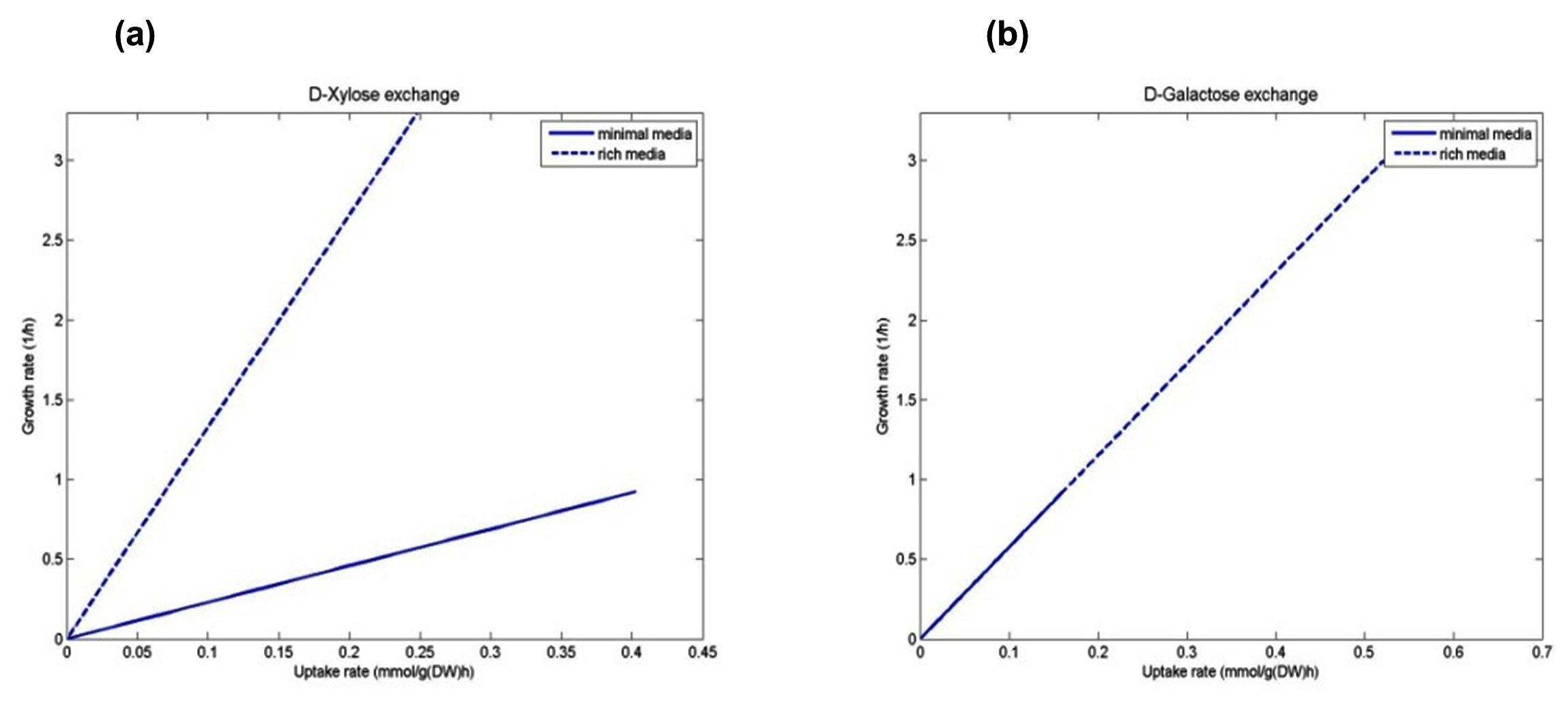

Supplement: Figure S1 — The predicted growth rate of the D-xylose (a) and D-galactose (b) biosensors, as a function of sensed chemical update rate, under both poor and rich media. As shown, the growth yield (represented by the slope of the curve) of the D-xylose biosensor significantly differ between poor and rich spent media. Hence if the D-xylose biosensor is grown after a minimal medium is supplemented to the spent medium, the potential secretion of additional metabolites by the producer strain may affect its biomass yield, leading to inaccurate estimation of the chemical concentration (an accurate estimation of D-xylose concentration can still be achieved by growing the biosensor after adding all nutrients from a rich medium (other than D-xylose, of course) to the spent. On the other hand, the D-galactose biosensor is predicted to have the same biomass yield in both poor and rich medium, facilitating the direct quantification of chemical concentration also in poor medium. (TIF) [file pone.0016274.s001.tif]
